# Three-Dimensional Reconstruction of Pacemaker Lead Trajectory From Orthogonal Chest X-Rays: A Proof of Concept

**DOI:** 10.7759/cureus.20807

**Published:** 2021-12-29

**Authors:** Akinori Higaki, Yoshitaka Kawada, Go Hiasa, Tadakatsu Yamada, Hideki Okayama

**Affiliations:** 1 Department of Cardiology, Ehime Prefectural Central Hospital, Matsuyama, JPN

**Keywords:** chest x-ray (cx-ray), pacemaker lead migration, virtual reality simulation, three-dimensional imaging, cardiac implantable electronic device (cied)

## Abstract

Understanding the lead trajectory is important in preventing complications after cardiac rhythm device implantation. In this report, we sought to reconstruct the three-dimensional (3D) shape of a pacing lead from radiographs taken at 90-degree angles. All image data were obtained from a 65-year-old male patient, who underwent pacemaker implantation at our hospital due to third-degree atrioventricular block in 2016. Both frontal and lateral chest X-rays were taken just after the device implantation (supine position) and on the post-procedural day 1 (upright position), respectively. Fluorine-18-fluorodeoxyglucose positron emission tomography/CT was performed 75 days after the pacemaker implantation for the diagnosis of cardiac sarcoidosis. Contours of the ventricular leads were manually traced in each X-ray image and saved as Scalable Vector Format (SVG) files using the GNU Image Manipulation Program (GIMP). The 3D reconstruction was performed on Blender 2.93, which is an open-source computer graphics software. The lead trajectory could be reconstructed from bidirectional radiographs, which may allow for further investigation of the 3D shape change of the pacemaker leads.

## Introduction

Understanding the lead trajectory is important in preventing complications after cardiac rhythm device implantation [1,2]. Since pacemaker implantation is usually performed in the supine position, the spatial relationship between cardiac chamber and device leads changes when the patient stands upright [3]. However, there is scarce research on the effect of postural changes in the deformation of pacemaker leads. One of the reasons that makes the analysis difficult is that the three-dimensional (3D) shape of the lead cannot be ascertained from a simple radiograph. A 3D construction is possible using computed tomography (CT), but it is difficult to perform the examination in a standing position. Currently, a number of studies have reported the feasibility of 3D reconstruction of coronary arteries from multiple angiographic projections in the field of interventional cardiology [4-6]. In this study, we propose a method to reconstruct the 3D shape of a pacing lead from radiographs taken at 90-degree angles. Reconstructed lead trajectory was then compared to the assumed true trajectory, which was extracted from the same patient’s CT images for validation purposes.

## Technical report

A case presentation and the image acquisition

All image data were obtained from a 65-year-old male patient, who underwent pacemaker implantation at our hospital due to third-degree atrioventricular block in 2016. Both frontal and lateral chest X-rays were taken just after the device implantation (supine position) and on the post-procedural day 1 (upright position), respectively. Fluorine-18-fluorodeoxyglucose positron emission tomography/CT was performed 75 days after the pacemaker implantation for the diagnosis of cardiac sarcoidosis.

Image processing

**Figure 1:**
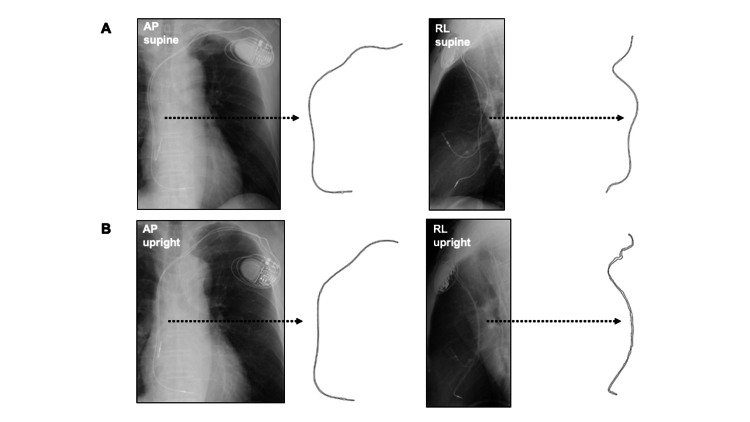
Manual tracing of the trajectory of ventricular leads. Contours of the right ventricular trajectories were manually traced on the chest radiographs and saved in SVG format. Panels A and B indicate contour extraction in the supine position and upright position, respectively, as shown with dotted arrows. SVG, Scalable Vector Format

As shown in Figure 1, contours of the ventricular leads were manually traced in each X-ray image and saved as Scalable Vector Format (SVG) files using the GNU Image Manipulation Program (GIMP). A Gaussian filter was used to help visually identify the lead contours.

**Figure 2:**
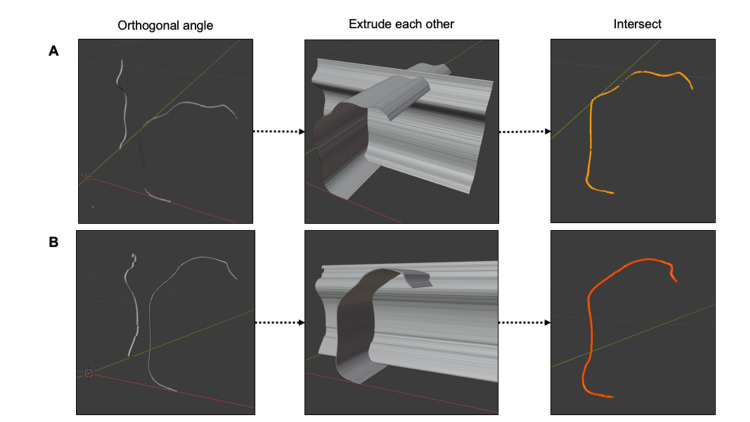
Three-dimensional reconstruction of ventricular leads from orthogonal images. Lead trajectories were reconstructed by taking intersects between extruded faces of orthogonal images. Panels A and B show the lead reconstruction in the supine and upright positions, respectively. Dotted arrows indicate the workflow.

The 3D reconstruction was performed on Blender 2.93, which is an open-source computer graphics software. First, the contour lines of the paired ventricular leads that form a 90-degree angle were imported into the software. After placing the faces on the contours, we used an "extrude" function to increase the thickness perpendicular to the surfaces. Next, a Boolean modifier was used to extract the intersection area between the volumes projected from two directions. The extracted trajectory was saved in STL format for further analysis. The schematic workflow of the 3D reconstruction is shown in Figure 2.

As for 3D construction from CT images, we used SYNAPSE VINCENT (Fujifilm Corp., Tokyo, Japan), embedded in our electronic medical record system. Using an automatic extraction algorithm based on CT values, the implanted devices were collectively recognized. After that, the generator and atrial lead were manually deleted. The shape data of the isolated ventricular lead were exported as STL file format.

Shape comparison with distance analysis

**Figure 3:**
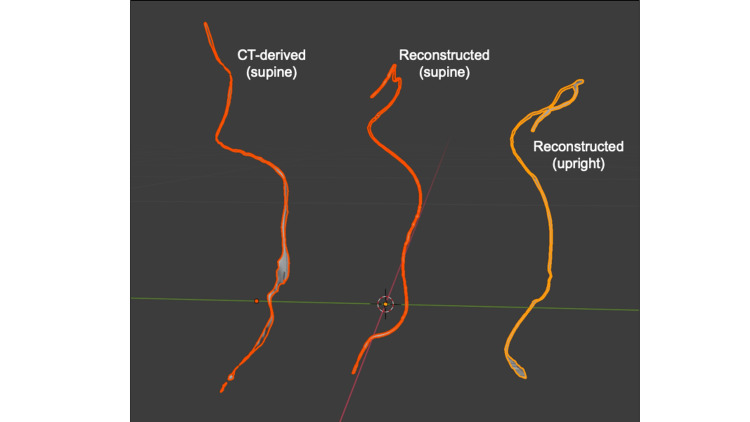
Visual comparison of the reconstructed lead trajectories. CT-derived and two reconstructed lead trajectories were aligned in the Unity workspace. The green line indicates the frontal axis, and the red line indicates the lateral axis.

Figure 3 shows the visual comparison of spatially aligned three shapes of the pacemaker lead, namely 3D construction from CT, reconstructed from X-ray at supine position and reconstructed from X-ray at standing position. Compared to the trajectory reconstructed from X-ray at upright position, the rest two trajectories, of which the image sources were obtained at supine position, show a visually similar shape.

**Figure 4:**
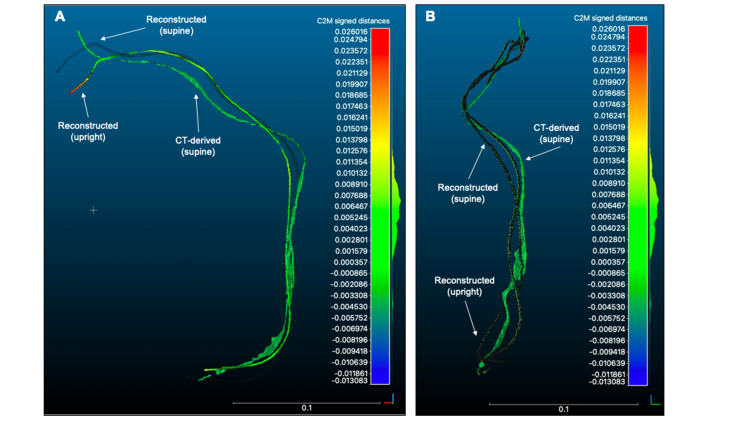
Quantitative assessment of distance between lead trajectories. Three-dimensional registration was conducted between reconstructed leads and CT-derived lead trajectory using the ICP method. Panels A and B show the final positions of the registered trajectories in frontal and lateral views, respectively. Distance to the reference trajectory is indicated by a color bar with metrics. ICP, interactive closest point

CloudCompare 2.12 (https://www.danielgm.net/cc/), which is an open-source software for 3D analysis, was used to quantitatively assess the similarity among three models, according to the previous literature [7]. First, reconstructed lead models were respectively aligned to the CT-derived lead trajectory (reference) using an “interactive closest point (ICP) registration” tool. The mean Euclidean distance between each trajectory and the reference model was automatically computed by the “Cloud/Mesh Distance” tool. When registered to the referential CT-derived lead trajectory, the reconstructed trajectory from supine X-rays has a shorter average distance (mm) to the reference than that of the model made from upright X-rays (5.35±5.51 vs 7.62±7.56). The registered lead trajectories are visualized in Figure 4. The mean distance between reconstructed leads (difference between supine and upright) was 3.25±9.20 after ICP registration.

## Discussion

Computer vision technology can derive a lot of information from simple chest X-ray images [8]. In this technical report, we have demonstrated a proof-of-concept that the trajectory of the right ventricular pacemaker lead could be reconstructed from chest radiography taken at 90-degree angles. This reconstruction method allows us to quantitatively analyze the shape change of the lead wire due to the patient's posture. As we demonstrated, the reconstructed lead shape obtained in the supine position was similar to that reconstructed from CT images, which were also scanned in the supine position. We are aware that our method has several limitations. First of all, this is just a case report to show the feasibility of 3D reconstruction from chest radiographs and therefore needed to be validated with other samples. Our reconstruction method is based on the assumption that the angle between the front and side radiographs is exactly 90 degrees and that the cardiac and respiratory cycles are the same at the time of imaging. In this report, we set the CT-derived 3D model as a reference. However, since the CT is performed in a raised arm position, the lead shape should be changed due to the traction. Finally, we have yet to demonstrate the clinical significance of this approach. Previously, lead displacement due to large breast size in a woman has been reported [9]. Therefore, our future goal is to identify clinical parameters to predict the amount change of lead shape due to postural changes.

## Conclusions

The lead trajectory can be reconstructed from bidirectional radiographs, which may allow for further investigation of the 3D shape change of the pacemaker leads. However, the validity of this method should be tested with a larger sample size.
